# Quantification of plasma phosphorylated tau to use as a biomarker for brain Alzheimer pathology: pilot case-control studies including patients with Alzheimer’s disease and down syndrome

**DOI:** 10.1186/s13024-017-0206-8

**Published:** 2017-09-04

**Authors:** Harutsugu Tatebe, Takashi Kasai, Takuma Ohmichi, Yusuke Kishi, Tomoshi Kakeya, Masaaki Waragai, Masaki Kondo, David Allsop, Takahiko Tokuda

**Affiliations:** 1 Department of Neurology, Kyoto Prefectural University of Medicine, Kyoto, 602-0841 Japan; 2 Department of Zaitaku (Homecare) Medicine, Kyoto Prefectural University of Medicine, Kyoto, 602-0841 Japan; 3 Strategic Marketing Division, SCRUM Inc, Tokyo, 130-0021 Japan; 4 Department of Neurology, Higashi Matsudo Municipal Hospital, Matsudo, 270-2222 Japan; 5 Division of Biomedical and Life Sciences, Faculty of Health and Medicine, Lancaster University, Lancaster, LA1 4YQ UK; 6 Department of Molecular Pathobiology of Brain Diseases, Kyoto Prefectural University of Medicine, Kyoto, 602-0841 Japan

**Keywords:** Plasma biomarker, Tau phosphorylated at threonine 181 (p-tau181), Simoa, Alzheimer’s disease, Down syndrome

## Abstract

**Background:**

There is still a substantial unmet need for less invasive and lower-cost blood-based biomarkers to detect brain Alzheimer’s disease (AD) pathology. This study is aimed to determine whether quantification of plasma tau phosphorylated at threonine 181 (p-tau181) is informative in the diagnosis of AD.

**Methods:**

We have developed a novel ultrasensitive immunoassay to quantify plasma p-tau181, and measured the levels of plasma p-tau181 in three cohorts.

**Results:**

In the first cohort composed of 20 AD patients and 15 age-matched controls, the plasma levels of p-tau181 were significantly higher in the AD patients than those in the controls (0.171 ± 0.166 pg/ml in AD versus 0.0405 ± 0.0756 pg/ml in controls, *p* = 0.0039). The percentage of the subjects whose levels of plasma p-tau181 exceeded the cut-off value (0.0921 pg/ml) was significantly higher in the AD group compared with the control group (60% in AD versus 16.7% in controls, *p* = 0.0090). In the second cohort composed of 20 patients with Down syndrome (DS) and 22 age-matched controls, the plasma concentrations of p-tau181 were significantly higher in the DS group (0.767 ± 1.26 pg/ml in DS versus 0.0415 ± 0.0710 pg/ml in controls, *p* = 0.0313). There was a significant correlation between the plasma levels of p-tau181 and age in the DS group (R^2^ = 0.4451, *p* = 0.0013). All of the DS individuals showing an extremely high concentration of plasma p-tau181 (> 1.0 pg/ml) were older than the age of 40. In the third cohort composed of 8 AD patients and 3 patients with other neurological diseases, the levels of plasma p-tau181 significantly correlated with those of CSF p-tau181 (R^2^ = 0.4525, *p* = 0.023).

**Conclusions:**

We report for the first time quantitative data on the plasma levels of p-tau181 in controls and patients with AD and DS, and these data suggest that the plasma p-tau181 is a promising blood biomarker for brain AD pathology. This exploratory pilot study warrants further large-scale and well-controlled studies to validate the usefulness of plasma p-tau181 as an urgently needed surrogate marker for the diagnosis and disease progression of AD.

**Electronic supplementary material:**

The online version of this article (doi:10.1186/s13024-017-0206-8) contains supplementary material, which is available to authorized users.

## Background

Alzheimer’s disease (AD) is the most common cause of dementia and one of the major medical problems to be resolved throughout the world. The pathognomonic brain pathologies characteristic of AD consist of senile plaques containing amyloid β (Aβ) peptide, and neurofibrillary tangles (NFT) composed of hyperphosphorylated tau protein. The same pathological changes are also present in the brains of aged people with Down syndrome (DS) [1] and so adults with DS can be regarded as a cohort of preclinical AD [2]. Accurate and sensitive biomarkers are needed urgently to aid in the diagnosis of AD, especially in those with preclinical AD, not least to facilitate the development of new disease-modifying treatments. Numerous previous studies searching for useful AD-biomarkers have reported the value of measuring levels of Aβ1–42, total (t-tau) and phosphorylated tau (p-tau) in cerebrospinal fluid (CSF), or visualizing plaques and tangles by means of positron emission tomography (PET) brain imaging using specific probes for Aβ or tau [3–6]. The current consensus is that these CSF and PET biomarkers can differentiate AD from normal aging and from other dementing disorders, and changes in these biomarkers can also facilitate detection of AD many years before any overt clinical symptoms become clear [7–10]. However, these CSF and PET biomarkers are, respectively, invasive and costly, and so they have not been utilized widely in routine clinical practice. Thus, there is still a substantial unmet need for less invasive and lower-cost alternatives, particularly for high-throughput screening of people at risk of developing AD. For this reason, there have been many studies of blood-based molecular markers, including plasma Aβ species, as a potential alternative and less invasive method for the diagnosis of AD. However, the reported results have been very contradictory [11–15] and so the prevailing view is that AD (or preclinical AD) cannot be distinguished from controls without AD pathology on the basis of plasma levels of Aβ40 and Aβ42 [16].

In the last few years a new method has become available for measuring levels of plasma t-tau by using an ultrasensitive digital enzyme-linked immunosorbent assay (ELISA) technique, and this has been studied as a potential diagnostic biomarker for AD. However, a substantial overlap was found in levels of plasma t-tau between patients with AD and age-matched controls [17]. Here, we report the development of a new assay using this type of ultrasensitive technique to quantify plasma p-tau phosphorylated at threonine 181 (p-tau181), instead of t-tau. There are no previous reports on quantification of plasma p-tau181 in patients with AD or in those with DS. In this small-scale pilot study, we have quantified levels of plasma p-tau181 in patients with AD and DS as well as control cases, and have compared levels between these groups.

## Methods

### Study design, subject characterization, and sample collection

To determine usefulness of p-tau181 for the diagnosis of AD pathology, we measured levels of p-tau181 in plasma and CSF samples obtained from 3 cohorts; 1) plasma samples obtained from 20 Caucasian patients with AD (ages 60–89, mean ± SD of 77.4 ± 7.7) and 15 age-matched Caucasian controls (ages 71–84, mean ± SD of 76.3 ± 3.2) (purchased from ProteoGenex, Inc., CA, USA via KAC Co. Ltd., Tokyo, Japan), 2) plasma samples from 20 patients with DS (ages 19–57, mean ± SD of 34.0 ± 11.5) and 22 age-matched healthy controls (ages 14–56, mean ± SD of 37.4 ± 12.0) recruited at outpatient clinic of Department of Neurology, Kyoto Prefectural University of Medicine, Kyoto, Japan and 3) plasma and CSF samples obtained simultaneously from 8 patients with AD and other neurological diseases (1 Parkinson’s disease (PD), 2 vascular dementia (VaD)) recruited at outpatient clinic of Department of Neurology, Higashi Matsudo Municipal Hospital, Chiba, Japan.

In the cohort 2, we enrolled 20 adult patients with DS from the registration for DS in Kyoto Prefectural University of Medicine and Hananoki Medical Welfare Center, from February 2013 to January 2017. Plasma of 22 age-matched healthy controls was obtained from another registration of Kyoto Prefectural University of Medicine during the aforementioned period. Social maturity in the DS patients was estimated as ‘social ages’ using the social maturity scale revised (S-M) (Nihonbunkakagakusha, Tokyo), which is a social maturity scale developed for the Japanese based on the Vineland Social Maturity Scale [18]. In 6 out of 20 patients with DS, ‘social ages’ were longitudinally evaluated twice, approximately 1 year apart, and calculated the changes of the social ages of the patient during that period (named as Δsocial ages). Brain Aβ-amyloid burden was evaluated with positron emission tomography (PET) using *N*-methyl-[11C]-2-(4′-methylamino-phenyl)-6-hydroxy-benzothiazole ([11C]PiB) in 6 DS patients as described previously [19]. A mean cortical PiB retention measurement was also computed across cortical regions of interest (ROIs) without including the cerebellum. The PiB retention outcomes were evaluated based on the standardized uptake value (SUV) measures. The SUVs were determined over 50–70 min post injection intervals and normalized to injected dose and body mass. SUV ratio (SUVR) was generated using the cerebellum as reference. In the cohort 3, patients were diagnosed based on clinical and neuroimaging findings; patients with AD were diagnosed using the National Institute of Neurological and Communication Disorders and Stroke-Alzheimer’s Disease and Related Disorders Association criteria for probable AD [20]; those with VaD were diagnosed according to NINDS (the National Institute of Neurological Disorders and Stroke)-AIREN (Association Internationale pour la Recherche et l’Enseignement en Neurosciences) clinical criteria for the diagnosis of VaD [21]; a patient with PD was diagnosed according to the UK PD Society Brain Bank criteria [22].

In the cohort 2 and 3, plasma samples were taken through venous puncture, and a total of 8 ml of blood was collected in EDTA-containing tubes. After collection, plasma was separated by centrifugation for 15 min at 2000 g and distributed in polypropylene vials, then stored at −80 °C until analysis. In the cohort 3, plasma and CSF samples were simultaneously taken. The CSF samples were centrifuged for 10 min at 400 g, aliquoted and stored at −80 °C, the plasma samples were processed as described above.

### Ethics, consent and permissions

All subjects including patients with DS provided written informed consent to participate in the study, which was approved by the University Ethics Committee (Kyoto Prefectural University of Medicine, Kyoto, Japan; the reference number RBMR-C-1027-2). The study procedures were designed and performed in accordance with the Declaration of Helsinki.

### Immunoassay protocols

For the CSF samples of cohort 3, the levels of CSF p-tau181 were measured using INNOTEST® PHOSPHO-TAU(181P) (FUJIREBIO Inc., Tokyo, Japan) according to the manufacturer’s instruction. The plasma levels of p-tau181 were analyzed with a novel ultrasensitive immunoassay specific for p-tau181 using digital array technology [23, 24]. To develop this novel p-tau181 immunoassay, we modified the Human Total Tau kit (Simoa™ Tau 2.0 Kit, Quanterix, Lexington, MA) on the Simoa HD-1 analyzer (Quanterix). This kit is an updated version of the assay reported previously [25] that uses a monoclonal capture antibody that reacts with a linear epitope in the midregion of all tau isoforms and a detection antibody that reacts with an epitope in the N-terminal region of t-tau. Instead of this detection antibody against t-tau, we employed anti-human PHF-tau monoclonal antibody AT270 (Thermo Fisher Scientific, Rockford, IL, USA) as the detection antibody for our novel p-tau181 immunoassay. This set of capture and detection antibodies specifically reacts with p-tau181 without reacting with unphosphorylated tau [26]. The other immunoassay reagents used in our p-tau181 assay were the same as those in the Simoa™ Tau 2.0 Kit, except for the calibrator to make the standard curve for the assay. We used Hu Tau [pT181] Standard in Human Tau [pT181] phosphoELISA™ ELISA kit (Invitrogen, Thermo Fisher Scientific) as the calibrator for our p-tau181 assay. The standard curve for our p-ta181 assay was carried out with duplicate measurements using the Hu Tau [pT181] Standard at different concentrations (0, 0.039, 0.15, 0.625, 2.5 and 10 pg/ml) of the p-tau181 protein diluted in Tau 2.0 Sample Diluent (contained in Simoa™ Tau 2.0 Kit). Regarding the assay procedure for plasma p-tau181, we followed that of the Simoa™ Tau 2.0 Kit except for changing the detection antibody and the calibrator as mentioned above. All plasma samples were diluted 4 times with the Tau 2.0 Sample Diluent prior to the assays, to minimize matrix effects. To eliminate inter-assay variability as a confounding factor, all plasma samples belonging to the same cohort were run in duplicate on the same day with the same lot of standards. The relative concentration estimates of plasma p-tau181 were calculated according to the standard curve. We assumed the levels of plasma p-tau to be 0 in samples in which we could not detect p-tau with our ultrasensitive assay in order to compare the patient and control groups.

The details of the procedures for method validation of our novel p-tau181 immunoassay are described in Additional file 1.

### Statistical analysis

Regarding differences between the patients with AD or DS and controls, the groups were compared using Mann–Whitney U test. Tau elevation profiles were also analyzed for area-under-the-curve (AUC) and receiver operating characteristics (ROC) in the cohort 1 (AD and controls). We tested associations between the levels of plasma p-tau181 and demographic factors in the cohort 2 (patients with DS and controls) as well as associations between levels of plasma and CSF p-tau181 in the cohort 3 (patients with DS and other neurological diseases) using Spearman correlation. All analyses were carried out using GraphPad Prism software (version 6.0, GraphPad software, San Diego, USA). The level of significance was set at *p* < 0.05. The characteristics of the patients in the cohort 3, including clinically diagnosed patients with AD (*n* = 8), VaD.

## Results

### Patient characteristics

Cohort 1 was a set of purchased samples consisting of plasma from 20 clinically diagnosed patients with AD and 15 controls (Table 1). Mean ages were matched for these two groups (mean ± standard deviation (SD: range), AD: 77.4 ± 7.7 (60–89), 76.3 ± 3.2 (71–84)).

**Table 1:** Characteristics of patients with AD and controls of cohort 1

Diagnosis	Number	M/F	Ages Mean ± SD (range)	MMSE scores Mean ± SD (range)
AD	20	8/12	77.4 ± 7.7 (60–89)	12.8 ± 5.0 (2–19)
Control	15	1/14	76.3 ± 3.2 (71–84)	N/A

M/F Male/female, AD Alzheimer’s disease, MMSE Mini-mental state examination, N/A not available

Cohort 2 consisted of 20 patients with DS (Table 2) and 22 healthy controls, and mean ages were matched for these two groups as mentioned above. Estimated social ages (18 out of 20 patients), the changes of social age during the 1-year follow-up period (6 out of 20), and the mean cortical SUVR values in PIB-PET study of the DS patients are shown in Table 2. Cohort 3 consisted of 8 AD, 2 VaD and 1 PD patients (Table 3), whose CSF and plasma samples were collected simultaneously in each patient to determine the correlation between CSF and plasma levels of p-tau181 as measured with our novel immunoassay.

**Table 2:** Characteristics of patients with DS of the cohort 2

Case	Sex	Age (years)	Social Age^a^ (years), BL	ΔSocial Age^b^ (years)	Mean cortical SUVR (PiB-PET)	Plasma p-tau181 (pg/ml)
1	M	36	8.75	1.10	1.553	0.0000
2	F	26	4.17	N/A	N/A	0.0000
3	F	19	4.50	2.00	1.400	0.0000
4	M	26	4.92	0.19	1.536	0.0000
5	F	22	7.42	−0.10	1.142	0.0000
6	F	31	N/A	N/A	N/A	0.0000
7	F	24	10.0	N/A	N/A	0.1494
8	F	25	7.00	N/A	N/A	0.0000
9	M	48	4.42	N/A	N/A	0.2040
10	M	25	7.08	−1.00	1.578	0.1408
11	F	45	1.92	N/A	N/A	2.0731
12	M	26	6.50	−1.10	N/A	0.2787
13	M	57	4.08	N/A	N/A	1.3516
14	M	31	2.33	N/A	N/A	0.2382
15	F	55	2.42	N/A	1.828	4.0296
16	F	42	4.17	N/A	N/A	2.7619
17	M	43	6.08	N/A	N/A	3.3685
18	M	25	N/A	N/A	N/A	0.3256
19	M	29	4.67	N/A	N/A	0.4112
20	F	45	8.33	N/A	N/A	0.0000

BL Baseline, SUVR standardized uptake value ratio, N/A not available

^a^Social ages were estimated using social maturity scale revised (S-M). Although data were calculated as units of years and months on this buttery [18], we recalculated those into unit of years for statistical analysis. Social ages in this figure were represented as unit of years

^b^“ΔSocial Age” represents the changes of the social age in individual DS patients who can be evaluated twice, approximately 1 year apart

**Table 3:** Characteristics of subjects of the cohort 3

Case	Sex	Age (years)	Clinical diagnosis	MMSE	CSF p-tau181 (pg/ml)	Plasma p-tau181 (pg/ml)
1	M	86	AD	17	63.5	0.1057
2	F	70	AD	23	96.0	0.3785
3	F	88	AD	20	51.7	0.1063
4	F	86	VaD	18	39.3	0.0783
5	F	75	AD	18	103.0	0.2774
6	M	76	AD	5	117.0	0.1797
7	F	76	AD	4	88.0	0.1057
8	F	85	AD	12	57.0	0.1162
9	F	68	PD	30	28.8	0.0629
10	F	82	VaD	18	71.8	0.0763
11	F	76	AD	24	67.6	0.1636

MMSE Mini-mental state examination, AD Alzheimer’s disease, VaD Vascular dementia, PD Parkinson’s disease

### Standard curve for the novel p-tau181 immunoassay and assay method validation

**Fig. 1:**
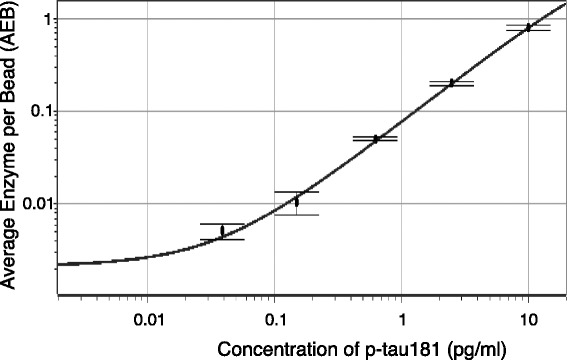
Standard curve for the plasma p-tau181 immunoassay (ultrasensitive digital array technology, Simoa™ system, Quanterix). Data represent the mean ± SD of duplicate readings. The goodness of fit was 0.9999. The limit of detection of the assay is 0.0090 pg/ml

Figure 1 shows the standard curve for our novel p-tau181 immunoassay, demonstrating that p-tau181 was detected with high sensitivity. The goodness of fit was 0.9999. The repeatability of the standard curve was determined in separate experiments (Additional file 1), and showed permissible repeatability of the standard curve with CV values of assay signal intensities (quantified by units of average number of enzyme labels per bead (AEB) in Simoa assay) such as 22.0, 11.3, 8.5, and 2.8% for standard p-tau solutions of 0, 0.039, 0.15 and 0.625 pg/ml, respectively. The limit of detection (LOD) of the assay, which requires 50 μl of plasma, is 0.0090 pg/ml (determined as described in Additional file 1). The LOD of our novel p-tau assay is accordingly ~1000-fold more sensitive than conventional ELISAs, for example, the above-mentioned INNOTEST® PHOSPHO-TAU(181P) (LOD = 13 pg/ml). Intra-assay precision was determined (Additional file 1), and it was robust with coefficient of variations (CVs) between 2.8 and 11.2% (Additional file 2). To eliminate inter-assay variability, all plasma samples of the same cohort were analyzed on the same day. In quality control experiments (Additional file 1), mean and CV values of the plasma p-tau were calculated in each sample (*n* = 6), which determined CV values ranging 3.8 to 10.4% (Additional file 3). In spike recovery and parallelism experiments (Additional file 1), the mean recovery rates were calculated to be 121.6 ± 3.5% after subtraction of the endogenous p-tau concentration. Parallelism of serially diluted plasma samples is also demonstrated in Additional file 4, which showed that plasma samples diluted 1:4 to 1:16 gave reliable results.

### Comparison of the plasma levels of p-tau181 in cohort 1

**Fig. 2:**
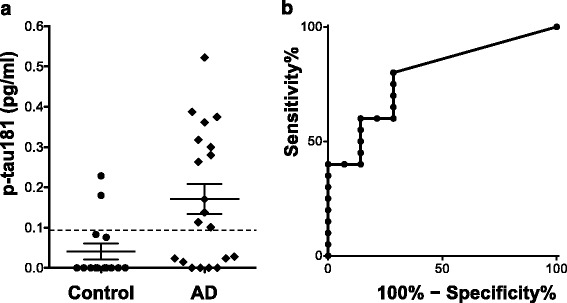
**a** Plots for the concentrations of plasma p-tau181 in the control patients (*n* = 15) and the clinically diagnosed patients with AD (*n* = 20) of Cohort 1. The solid lines represent the mean value ± standard errors (SE) of the concentrations of each group. The concentration of plasma p-tau181 in the AD group was significantly higher than that in the age-matched control subjects (*p* = 0.0039, Mann–Whitney U test). The dashed line corresponds to the cut-off value of the plasma p-tau181 to discriminate those two groups (0.0921 pg/ml). **b** ROC analysis of the levels of plasma p-tau181 for the discrimination between AD and control groups (AUC = 0.786, sensitivity = 60.0%, specificity = 85.7%)

The plasma levels of p-tau181 were significantly higher in the AD group (mean ± SD 0.171 ± 0.166 pg/ml, 95% confidence interval (95% CI) of the mean 0.0934–0.248, *n* = 20) than those in the age-matched controls (0.0405 ± 0.0756 pg/ml, 95% CI of the mean − 0.00311 – 0.0842, *n* = 15) (*p* = 0.0039, Mann–Whitney U test; Fig. 2a). Figure 2b shows the ROC curve for the classification of patients with AD and controls based on the levels of plasma p-tau181 measured in Cohort 1. The AUC for the ROC analysis was 0.786. From the ROC curve analysis, when the cut-off value of the plasma p-tau181 used to discriminate those two groups was set at 0.0921 pg/ml, we obtained the highest likelihood ratio of the classification belonging to the AD or control group. Among the 20 patients with AD and 15 controls, there were 12 patients (60.0%) and 2 controls (16.7%) with levels of plasma p-tau181 higher than this cut-off value (i.e. more than 0.0921 pg/ml, Table 1, Fig. 2a). Thus, the percentage of the subjects whose levels of plasma p-tau181 exceeded the cut-off value was significantly higher in the AD group compared with the control group (*p* = 0.0090, Fisher’s exact test).

### Plasma levels of p-tau181 in patients with DS

**Fig. 3:**
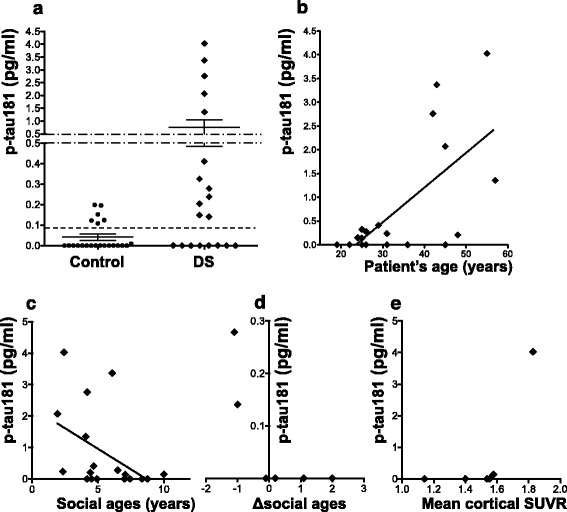
**a** Plots for the concentrations of plasma p-tau181 in the control patients (*n* = 22) and the clinically diagnosed patients with DS (*n* = 20) of Cohort 2. The solid lines represent the mean value ± standard errors (SE) of the concentrations of each group. The concentration of plasma p-tau181 in the DS group was significantly higher than that in the age-matched controls (*p* = 0.0313, Mann–Whitney U test). The dashed line corresponds to the cut-off value of the plasma p-tau181 to discriminate AD from control (0.0921 pg/ml) derived from the ROC analysis of AD and control groups in Cohort 1 (Fig. 2a). **b** A scatter plot of the levels of plasma p-tau181 versus the patient age of the DS patients (*n* = 20) and a linear regression line for the correlation of those two parameters. There is a significant correlation between the levels of plasma p-tau181 and the age of the DS patients (R^2^ = 0.4451, *p* = 0.0013, Pearson correlation). **c** A scatter plot of the levels of plasma p-tau181 versus the social ages of the DS patients (*n* = 18) indexed by social maturity scale developed for the Japanese (S-M) [18] that represents the intellectual ability of each DS patient. The solid lines represent the linear regression line between those two parameters. The levels of plasma p-tau181 were weakly correlated negatively with the social ages of DS patients, but the correlation was not significant (*p* = 0.0563, *n* = 18). **d** A scatter plot of the levels of plasma p-tau181 versus the Δsocial ages of the DS patients (*n* = 6), which indicates the changes of the social ages of the patient during the ~1-year follow-up period. The larger negative values of Δsocial ages means the more cognitive decline the patient had. **e** A scatter plot of the levels of plasma p-tau181 versus the mean cortical SUVR in PiB-PET study of the DS patients (*n* = 6), which represents the severity of cerebral Aβ-amyloid burden

The plasma levels of p-tau181 were significantly higher in the DS group (mean ± SD 0.767 ± 1.26 pg/ml, 95% CI of the mean 0.178–1.355, *n* = 20) than those in the age-matched controls (0.0415 ± 0.0710 pg/ml, 95% CI of the mean 0.0100–0.0729, *n* = 22) (*p* = 0.0313, Mann–Whitney U test; Fig. 3a). When the cut-off value to discriminate those two groups was set to 0.0921 pg/ml, that is the value obtained for Cohort 1 (AD vs. Control), 12 out of 20 patients with DS (60.0%) and 6 out of 22 controls (27.3%) had levels of plasma p-ta181 higher than the cut-off value (i.e. more than 0.0921 pg/ml, Fig. 3a). The percentage of the subjects whose levels of plasma p-tau181 exceeded the cut-off value was significantly higher in the DS group compared with the control group (*p* = 0.0332, Fisher’s exact test).

Individuals with DS develop dementia at a much lower age, and also have a shorter life expectancy in general. Thus, age-matched controls mentioned above may not possibly be the right control group. As a result, we have made another comparison between the levels of plasma p-tau in the DS group and those in the controls for the AD group in our cohort 1. We found that there is still a significant difference in the plasma p-tau levels between those two groups (*p* = 0.0116, Mann–Whitney U test).

A significant correlation between the plasma levels of p-tau181 and age was observed in the DS group (R^2^ = 0.4451, *p* = 0.0013, Pearson correlation; Fig. 3b), but not in the control group (R^2^ = 0.0317, *p* = 0.4275, data not shown). All of the DS patients showing an extremely high concentration of plasma p-tau181 (> 1.0 pg/ml) were older than the age of 40 (Fig. 3b). Plasma p-tau181 levels in patients with DS were weakly, but not significantly, correlated negatively with their social ages (*p* = 0.0563, *n* = 18; Fig. 3c). In the DS patients whose social ages were longitudinally evaluated (*n* = 6; Table 2), 2 patients who exhibited a decrease in social age, suggesting that their cognitive function had started to decline, had abnormally high levels of p-tau181. Meanwhile, those whose social ages remained unchanged showed much lower values of plasma p-tau181 around 0 pg/ml (Fig. 3d).

We performed a PiB-PET study in 6 individuals with DS aged 19 to 55 years (Table 2). The level of plasma p-tau181 was increased in a patient of 55 years who had a clear positive Aβ-amyloid burden in the cerebral cortices (mean cortical SUVR = 1.828), while the levels of p-tau181 were lower (around 0 pg/ml) in the other patients whose mean cortical SUVRs were less than 1.6 (Fig. 3e).

### Correlation between the levels of p-tau181 in CSF and plasma

**Fig. 4:**
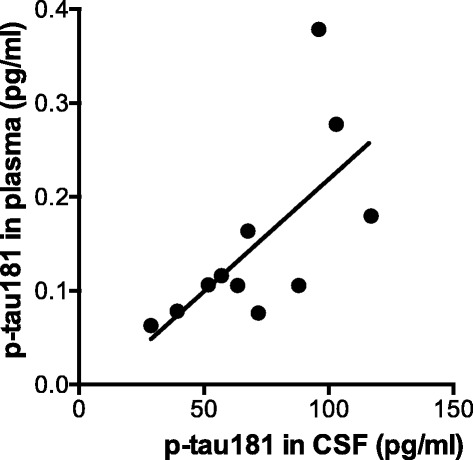
A scatter plot of the levels of plasma p-tau181 versus those of CSF p-tau181 in the patients of Cohort 3 (*n* = 11) and a linear regression line for the correlation of those two parameters. There is a significant correlation between the levels of plasma and CSF p-tau181 in this small cohort (R^2^ = 0.4525, *p* = 0.023, *n* = 11, Pearson correlation)

We next examined any correlation between the CSF and plasma levels of p-tau181 in the patients in Cohort 3 (*n* = 11) in which matched CSF and plasma samples were taken from each patient. In this small cohort, the levels of plasma p-tau181 significantly correlated with those of CSF p-tau181 (R^2^ = 0.4525, *p* = 0.023, *n* = 11, Pearson correlation; Fig. 4).

## Discussion

To our knowledge, this is the first study to report an immunoassay method that can specifically quantify the levels of p-tau181 in human plasma and to present quantitative data on the plasma levels of p-tau181 in patients with AD and DS as well as in control subjects. We used a recently developed digital array technology (Simoa™ system, Quanterix) that can be applied to development of unprecedented biomarkers for brain diseases due to its ultra-high sensitivity. Even in the control subjects of Cohorts 1 and 2, we were able to obtain values for plasma p-tau181 concentrations due to the ultra-high sensitivity of this assay to detect minute amounts of p-tau181 in human plasma. The determined mean levels of plasma p-tau181 in the control subjects were similar between Cohort 1 and Cohort 2 (mean ± SD 0.0405 ± 0.0756 pg/ml, 95% CI of the mean − 0.00311 – 0.0842 in the controls of Cohort 1; mean ± SD 0.0415 ± 0.0710 pg/ml, 95% CI of the mean 0.0100–0.0729 in those of Cohort 2), suggesting that our p-tau181 immunoassay is reliable. Ours is the first study to suggest that the plasma level of p-tau181 is a promising blood biomarker for brain AD pathology in patients with AD and DS. Although the AUC value (0.786) for the ROC analysis using Cohort 1 (AD vs. Control) was not so high, this could be mainly due to the nature of this pilot study which involved only a small number of subjects. As shown in Fig. 2a, discrimination between AD and controls based on the levels of plasma p-tau181 was fairly good when the cut-off value was set at 0.0921 pg/ml, because the percentage of subjects with levels of plasma p-tau181 above this cut-off was significantly higher in the AD group (60.0%) than the control group (16.7%) (*p* = 0.0090). We also confirmed a significant discrimination ability of the levels of plasma p-tau181 between the DS and control group in Cohort 2 by using the same cut-off value as for Cohort 1 (0.0921 pg/ml) (*p* = 0.0332, Fig. 3a). Interestingly, all of the DS patients who showed extremely high concentrations of plasma p-tau181 (> 1.0 pg/ml) were older than the age of 40, which is around the age when tau pathology begins to develop in the brains of DS individuals [1]. Regarding the relevance to brain AD pathology, among the 6 patients with DS who underwent PiB-PET, the level of plasma p-tau181 was not increased in the 5 patients whose mean cortical SUVRs were less than 1.6, but increased only in the patient with an obviously positive amyloid burden (mean cortical SUVR = 1.828) (Fig. 3e). Further, we consider that the levels of plasma p-tau181 might correlate with cognitive status in the DS cohort. Plasma p-tau181 levels in patients with DS had a tendency to correlate negatively with their social ages (Fig. 3c), and were increased only in the 2 patients who started to suffer from cognitive decline with a decrement in their social age during the ~1-year follow-up period (Fig. 3d).

Our aim was to develop a novel ultrasensitive assay for p-tau181 and to use it as a possible blood biomarker for the diagnosis of brain AD pathology. Although the value of the three core CSF biomarkers (Aβ42, t-tau, and p-tau) has been established in numerous studies, including meta-analyses that have used a huge number of subjects [3–6], these CSF biomarkers have serious limitations because of their invasiveness and the considerable care and skill needed to collect CSF samples, and so these markers have not found their way into routine clinical use. Regarding potential blood biomarkers for AD, plasma Aβ40 and/or Aβ42 have been studied, but contradictory results were reported [11–15]. Thus, at the present time, patients with AD pathology cannot be distinguished from controls by measuring the levels of plasma Aβ species [16]. Since t-tau is another reasonable candidate plasma biomarker for AD pathology, there have been several studies that have explored the levels of plasma t-tau in patients with AD and related disorders in comparison with healthy controls [17, 27–33]. Using a recently-developed ultrasensitive digital ELISA method and plasma samples obtained from large cohort studies, Zetterberg et al. reported levels of plasma t-tau in large numbers of patients with AD and controls [17, 28]. Because the overlap between normal aging and AD was large in their studies, they concluded that their results do not support plasma t-tau as an AD biomarker in individual people, despite group-level differences in plasma levels between those two groups. One possible reason for the failure of plasma Aβ species and t-tau as specific biomarkers for AD pathology is that these molecules can have a peripheral source outside of the brain, for example plasma Aβ species may originate from platelets [34, 35] and t-tau from peripheral nerves [36, 37]. We have focused on the development of a novel immunoassay to quantify plasma p-tau181, because high CSF t-tau is not only found in AD but also in other brain disorders with neuronal damage, while high CSF p-tau is more specific for brain AD pathology [5, 38, 39]. In our assay, we used an established set of capture and detection antibodies that have been validated already in previous studies, including large cohort studies such as the Alzheimer’s Disease Neuroimaging Initiative (ADNI) study; the capture antibody is the same as that used in the Simoa™ Tau 2.0 Kit (Quanterix) which can determine exact levels of plasma t-tau in samples from AD and control cases; the detection antibody (AT270) has been used in the international standard ELISA to quantify CSF p-tau181 (INNOTEST® PHOSPHO-TAU(181P), FUJIREBIO Inc.) in many previous studies [40, 41].

We also demonstrated a significant positive correlation between plasma and CSF p-tau181 levels in Cohort 3. This finding suggests that the level of plasma p-tau181 is likely to reflect the concentration of p-tau181 in the interstitial and extracellular compartments within the brain, and therefore p-tau181 in plasma should be of brain origin. Regarding the correlation of plasma and CSF levels of t-tau, a recent large-scale study demonstrated that the levels of t-tau in plasma were positively associated with those in CSF, but this association was weak and differed according to the examined cohort; the association was not significant in the ADNI cohort, but significant in the other cohort [17]. Considering this previous report and our results together, we consider that the differences between plasma-CSF correlations of p-tau181 and t-tau could mainly reflect their different sources from within and outside of the brain, as mentioned above; t-tau can have both a brain and peripheral origin [36, 37], whereas p-tau181 should be exclusively of brain origin [5, 38, 39].

This is the first study to quantify at an ultrasensitive level the amount of p-tau181 in human plasma and to show the value of this measure for the diagnosis of brain AD pathology, but it is not without limitations. Firstly, this is a small-scale exploratory pilot study, and the data presented are still preliminary. Thus, we need to confirm whether or not plasma p-tau181 will be useful as a diagnostic biomarker for brain AD pathology as well as a biomarker to evaluate disease severity in large-scale studies. Blood-based biomarkers are required for early/preclinical diagnosis and for selection of appropriate subjects for clinical trials of disease-modifying therapies, and we consider that plasma p-tau181 is a promising candidate for an AD blood biomarker. Another limitation is that we only used plasma and CSF samples from clinically diagnosed patients not proven by autopsy. It has been reported that more than 20% of patients diagnosed clinically and recruited by dementia specialists do not have any cerebral amyloid burden in amyloid PET studies [42], suggesting that the diagnosis of AD based only on clinical symptoms, without objective biomarkers, is flawed. In Cohort 1 of the present study, 8 out of 20 AD patients who had been diagnosed based on clinical symptoms showed levels of plasma p-tau181 lower than the cut-off value differentiating between AD and control groups (Fig. 2a). One of the reasons for this finding is the possibility that some of those patients with lower plasma p-tau181 could be non-AD dementia, misdiagnosed as AD. Supporting this possibility, in Cohort 2, all of the 7 DS patients aged over 40 (except a 45-year-old female), which is around the age when tau pathology starts in DS, showed substantially and extremely elevated levels of plasma p-tau181 compared to those of the controls (Fig. 3a).

## Conclusions

By using a newly developed ultrasensitive immunoassay, we report for the first time quantitative data on the plasma levels of p-tau181 in patients with AD and DS as well as control subjects. Our results provide highly valuable information that the plasma p-tau181 is a promising blood biomarker for the detection of brain AD pathology, since there is still a great unmet need for less invasive and lower-cost blood-based biomarkers to detect brain AD pathology. This small-scale pilot study warrants further large-scale and well-controlled studies with strict protocols, especially those including patients with AD who have brain AD pathologies proven by autopsy and/or PET studies for detection of cerebral amyloid and tau burden, to validate the usefulness of plasma p-tau181 as an urgently needed blood biomarker for the diagnosis and disease progression of AD.

## Additional files

Additional file 1: Supplementary methods. Description of data: Additional methods that describe procedures for method validation. (PDF 60 kb)

Additional file 2: Supplementary Table 1. Description of data: Results of intra-assay precision. (PDF 37 kb)

Additional file 3: Supplementary Table 2. Description of data: Results of inter-assay precision for quality control samples. (PDF 37 kb)

Additional file 4: Title of data: Supplementary Fig. 1. Description of data: Results of spike recovery and Parallelism. (PDF 197 kb)

## Abbreviations

[11C]PiB: *N*-methyl-[11C]-2-(4′-methylamino-phenyl)-6-hydroxy-benzothiazole
95% CI: 95% confidence interval
AD: Alzheimer’s disease;
ADNI: Alzheimer’s Disease Neuroimaging Initiative
AUC: area under the curve
BL: baseline
CSF: cerebrospinal fluid
CV: coefficient of variation
DS: Down syndrome
ELISA: enzyme-linked immunosorbent assay
LOD: the limit of detection
M/F: male/female
MMSE: mini-mental state examination
N/A: not available
NFT: neurofibrillary tangles
NINDS-AIREN: the National Institute of Neurological Disorders and Stroke-Association Internationale pour la Recherche et l’Enseignement en Neurosciences
PD: Parkinson’s disease
PET: positron emission tomography
p-tau181: tau phosphorylated at threonine 181
ROC: receiver operating characteristics
SD: standard deviation
SE: standard errors
S-M: social maturity scale revised
SUV: standardized uptake value
SUVR: standardized uptake value ratio
t-tau: total tau
VaD: vascular dementia
