# Supplementary material for: Quantification of plasma phosphorylated tau to use as a biomarker for brain Alzheimer pathology: pilot case-control studies including patients with Alzheimer’s disease and down syndrome
Source: Mol Neurodegener. 2017 Sep 4;12:63. doi: 10.1186/s13024-017-0206-8 (PMC5582385; doi:10.1186/s13024-017-0206-8)
Supplement: Supplementary file 1 — Description of data: Additional methods that describe procedures for method validation. (PDF 60 kb) [file 13024_2017_206_MOESM1_ESM.pdf]

Supplementary Methods

Procedures for method validation

1) Limit of detection (LOD)

We prepared 21 aliquots of blank sample (Tau 2.0 Sample Diluent, contained in Simoa^{TM} Tau 2.0 Kit), and measured "background" signals of our novel plasma p-tau immunoassay on the Simoa HD-1 analyzer (Quanterix). In Simoa, the measured signals are quantified by a common unit, namely average number of enzymes labels per bead (AEB). Then, the LOD of the assay was determined as an interpolated p-tau concentration derived from the mean plus 2.5 SD value of AEBs for the blank samples.

2) Intra-assay precision

Eighteen samples with different (high, moderate, or low) concentrations of p-tau were prepared for analysis of intra-assay precision, and measured the levels of p-tau in one experiment. Intra-assay precision was determined by calculating within-run coefficient of variation (CV) for those samples.

3) Inter-assay precision for quality controls and repeatability of the standard curve

We prepared 6 plasma samples with different concentrations of plasma p-tau from 4 patients with AD, 1 patient with DS and 1 control for quality control experiments, and measured the levels of

p-tau in those samples twice on different days. Inter-assay precision was determined by calculating CV between the runs for those samples.

To evaluate repeatability of the standard curve, we separately determined inter-assay precision by calculating CV of AEBs derived from standard solutions, used for making standard curves, with different concentrations (0, 0.039, 0.15 and 0.625 pg/ml) of p-tau standard (Hu Tau [pT181] Standard in Human Tau [pT181] phosphoELISA™ ELISA kit, Invitrogen, Thermo Fisher Scientific).

4) Spike recovery and parallelism

Three aliquots of the same plasma sample were prepared, and spiked with 0, 0.05 or 0.10 pg of p-tau standard respectively for spike recovery experiments. After subtraction of the endogenous p-tau concentration, the mean recovery rates were calculated. Next, those 3 solutions were serially diluted 4-fold and 16-fold with sample diluent for analysis starting with non-diluted solution as the highest concentration to evaluate parallelism.
