# Supplementary material for: Quantification of plasma phosphorylated tau to use as a biomarker for brain Alzheimer pathology: pilot case-control studies including patients with Alzheimer’s disease and down syndrome
Source: Mol Neurodegener. 2017 Sep 4;12:63. doi: 10.1186/s13024-017-0206-8 (PMC5582385; doi:10.1186/s13024-017-0206-8)
Supplement: Supplementary file 2 — Description of data: Results of intra-assay precision. (PDF 37 kb) [file 13024_2017_206_MOESM2_ESM.pdf]

Supplementary table 1. Intra-assay precision (n = 18)

	Sample 1	Sample 2	Sample 3
Mean (pg/ml)	0.0660	0.098	0.6613
SD	0.0074	0.0105	0.0183
CV(%)	11.2	10.7	2.8
