# Supplementary material for: Quantification of plasma phosphorylated tau to use as a biomarker for brain Alzheimer pathology: pilot case-control studies including patients with Alzheimer’s disease and down syndrome
Source: Mol Neurodegener. 2017 Sep 4;12:63. doi: 10.1186/s13024-017-0206-8 (PMC5582385; doi:10.1186/s13024-017-0206-8)
Supplement: Supplementary file 3 — Description of data: Results of inter-assay precision for quality control samples. (PDF 37 kb) [file 13024_2017_206_MOESM3_ESM.pdf]

**Supplementary table 2. Inter-assay precision for quality control samples (n = 12)**

| | Sample 1 | Sample 2 | Sample 3 | Sample 4 | Sample 5 | Sample 6 |
|---|---|---|---|---|---|---|
| Mean (pg/ml) | 0.1270 | 0.0721 | 0.0402 | 0.0188 | 0.0108 | 0.0102 |
| SD | 0.0048 | 0.0044 | 0.0034 | 0.0012 | 0.0011 | 0.0004 |
| CV(%) | 3.8 | 6.1 | 8.5 | 6.5 | 10.4 | 4.2 |
