# Supplementary material for: Quantification of plasma phosphorylated tau to use as a biomarker for brain Alzheimer pathology: pilot case-control studies including patients with Alzheimer’s disease and down syndrome
Source: Mol Neurodegener. 2017 Sep 4;12:63. doi: 10.1186/s13024-017-0206-8 (PMC5582385; doi:10.1186/s13024-017-0206-8)
Supplement: Supplementary file 4 — Description of data: Results of spike recovery and Parallelism. (PDF 197 kb) [file 13024_2017_206_MOESM4_ESM.pdf]

Supplementary Figure 1. Spike recovery and Parallelism

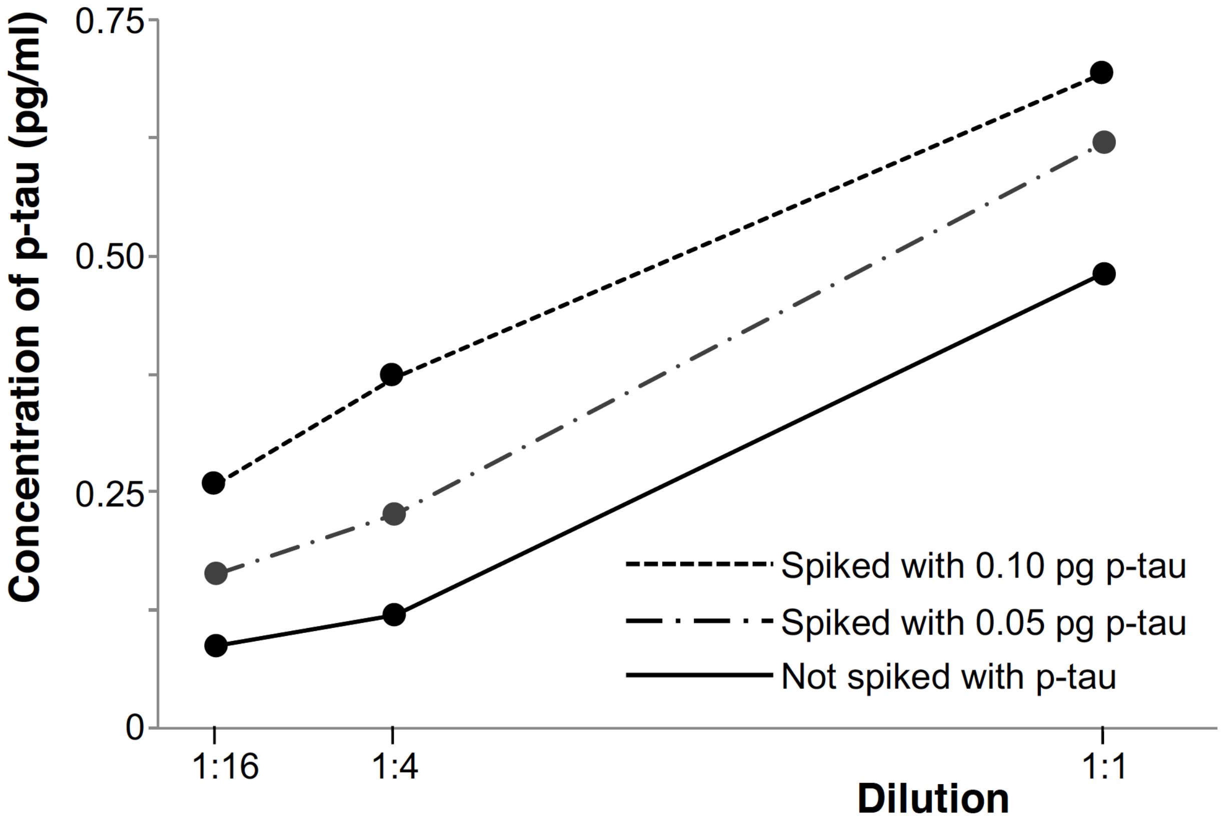

Figure legend for supplementary figure 1.

Three aliquots of the same plasma sample were spiked with 0 (solid line), 0.05 (one-dot chain line) or 0.10 (dotted line) pg of p-tau standard for a spike recovery experiment. Next, those 3 solutions were serially diluted 4-fold and 16-fold with sample diluent for analysis starting with non-diluted solution as the highest concentration to evaluate parallelism.
